# Exploring Gender Bias in AI for Personalized Medicine: Focus Group Study With Trans Community Members

**DOI:** 10.2196/72325

**Published:** 2025-07-29

**Authors:** Nataly Buslón, Davide Cirillo, Oriol Rios, Simón Perera del Rosario

**Affiliations:** 1 Social and Responsible Computing, Department of Engineering, Universitat Pompeu Fabra, Tànger, 122-140, Barcelona, 08018, Spain, +34 935 42 22 01; 2 Bioinfo4Women, Life Sciences Department, Barcelona Supercomputing Center, Barcelona, Spain; 3 Facultat de Ciències de l’Educació i Psicologia, Universitat Rovira i Virgili, Tarragona, Spain; 4 Association for Affective, Sexual and Gender Diversity in Science, Technology and Innovation (PRISMA), Pamplona, Spain; 5 Institute of Evolutionary Biology (UPF-CSIC), Department of Medicine and Life Sciences, Universitat Pompeu Fabra, Barcelona, Spain

**Keywords:** trans people, transgender medicine, precision medicine, artificial intelligence, artificial intelligence biases, health biases

## Abstract

**Background:**

This paper explores the perception and application of artificial intelligence (AI) for personalized medicine within the trans community, an often-overlooked demographic in the broader scope of precision medicine. Despite growing advancements in AI-driven health care solutions, little research has been dedicated to understanding how these technologies can be tailored to meet the unique health care needs of trans individuals. Addressing this gap is crucial for ensuring that precision medicine is genuinely inclusive and effective for all populations.

**Objective:**

This study aimed to identify the specific challenges, obstacles, and potential solutions associated with the deployment of AI technologies in the development of personalized medicine for trans people. This research emphasizes a trans-inclusive and multidisciplinary perspective, highlighting the importance of cultural competence and community engagement in the design and implementation of AI-driven health care solutions.

**Methods:**

A communicative methodology was applied in this study, prioritizing the active involvement of end-users and stakeholders through egalitarian dialogue that recognizes and values cultural intelligence. The methodological design included iterative consultations with trans community representatives to cocreate the research workflow and adapt data collection instruments accordingly. This participatory approach ensured that the perspectives and lived experiences of trans individuals were integral to the research process. Data collection was conducted through 3 focus groups with 16 trans adults, aimed at discussing the challenges, risks, and transformative potential of AI in precision medicine.

**Results:**

Analysis of the focus group discussions revealed several critical barriers impacting the integration of AI in personalized medicine for trans people, including concerns around data privacy, biases in algorithmic decision-making, and the lack of tailored health care data reflective of trans experiences. Participants expressed apprehensions about potential misdiagnoses or inappropriate treatments due to cisnormative data models. However, they also identified opportunities for AI to enhance health care outcomes, advocating for community-led data collection initiatives and improved algorithmic transparency. Proposed solutions included enhancing datasets with trans-specific health markers, incorporating community voices in AI development processes, and prioritizing ethical frameworks that respect gender diversity.

**Conclusions:**

This study underscores the necessity for a trans-inclusive approach to precision medicine, facilitated by AI technologies that are sensitive to the health care needs and lived realities of trans people. By addressing the identified challenges and adopting community-driven solutions, AI has the potential to bridge existing health care gaps and improve the quality of life for trans individuals. This research contributes to the growing discourse on equitable health care innovation, calling for more inclusive AI design practices that extend the benefits of precision medicine to marginalized communities.

## Introduction

Trans health care has recently gained visibility on the public agenda of international organizations such as the World Health Organization (WHO) and the European Commission. In 2023, the WHO began developing a guideline on the health of trans and gender-diverse people [1]. Similarly, the European LGBTIQ Equality Strategy (2020-2025) emphasizes the need to improve health training and health care of LGBTIQ+ (lesbian, gay, bisexual, transgender, intersex, queer, and other identities related to affective, sexual, and gender diversity) people, with particular attention to trans and intersex people [2]. This contrasts with previous approaches to trans health care, which were framed on a pathologizing perspective that can be traced back to at least the 1950s [3].

In this article, we use the word “trans” following the definition provided by the International Lesbian, Gay, Bisexual, Trans, and Intersex Association [4]:

“Trans” is an inclusive umbrella term referring to people whose gender identity and/or gender expression differ from the sex/gender they were assigned at birth. It may include, but is not limited to: people who identify as transsexual, transgender, transvestite/cross-dressing, androgyne, polygender, genderqueer, agender, gender variant, gender non-conforming, or with any other gender identity and/or expression which does not meet the societal and cultural expectations placed on gender identity.

Pivotal events in the history of health care for trans people include the multiple revisions of the analytical categories included in the *Diagnostic and Statistical Manual of Mental Disorders* (*DSM*) published by the American Psychiatric Association. These revisions aimed to enable access to necessary health care. The diagnosis for “transsexualism” first appeared in 1980 (*DSM-III*); in an effort to reduce stigma, it was replaced with “gender identity disorder in adults and adolescence” in 1994 (*DSM-IV*), and further replaced with “gender dysphoria” in 2013 (*DSM-V*) to focus on gender-related distress rather than pathologizing gender identities [5,6].

Although the *DSM-V* formulates explicitly that “gender nonconformity is not in itself a mental disorder,” controversy remains. Access to gender transition-related health care often still requires a formal diagnosis of gender dysphoria, which can lead to medical care denial, fear-driven malingering, self-medication, and eventually greater harm [7]. More broadly, the stigmatization of any diagnostic category remains a long-standing issue concerning the trans population [8].

In the past few years, the digital transformation of health care services has accelerated, driven in part by the demands of the COVID-19 pandemic [9]. Among the many aspects of this transformation, artificial intelligence (AI) has played a major role in enhancing medical and health care capabilities [10], although it is not exempt from biases in data collection and management affecting its accuracy, fairness, and generalizability [11]. Bias refers to a systematic error, made by a human (cognitive bias) or a machine (algorithmic bias), that results in unfair treatment of individuals or groups based on personal attributes such as sex, gender, age, or ethnicity. As a consequence, these biases can disproportionately harm marginalized and vulnerable communities. For example, gender bias in AI-based technologies can severely impact trans individuals, contributing to discrimination and adverse outcomes across various aspects of life [12]. Specifically, algorithmic biases often arise from limitations in training data, such as the underrepresentation or mislabeling of minority groups in health care datasets, leading to systematic misclassification and the reinforcement of existing social and health care inequalities [13,14]. These issues underscore critical ethical challenges related to fairness, accountability, and inclusivity in digital health systems, as emphasized by several global initiatives focused on protecting vulnerable communities (see Discussion).

AI is widely seen as a tool that could improve access to specialized health care, including telemedicine, robot-assisted surgery, and digital pathology [15]. However, it remains an open question whether AI can help accounting for the special needs of vulnerable groups who may face barriers to health care, or whether it might, instead, exacerbate existing inequalities. Specifically, would vulnerable and stigmatized groups such as the trans population benefit from digital health and AI? What is the current status of AI designed to improve the well-being of trans people? How is AI perceived within the trans community?

The scientific literature on AI and trans health care is relatively scarce. In the domain of gender-affirming voice care, an AI tool designed to improve voice perception has recently attracted interest from the trans community for tracking progress over time [16]. Likewise, a gender-neutral voice for digital assistants, called Q [17], has been developed in an attempt to reduce gender bias in such technologies. In cardiology, an AI system has been used to study variations in the electrocardiogram of individuals undergoing gender-affirming hormonal treatment [18]. AI tools for trans health care have also emerged in dermatology [19]. In surgery, although AI-based facial recognition typically relies on binary assumptions incurring the risk of misgendering individuals [20], this technology has recently been used to improve gender-typing of trans women after facial feminization surgery [21]. As for telemedicine, challenges for trans health care remain largely unassessed [22]; however, mobile health technology has shown the ability to increase uptake of HIV testing among young trans women [23] and engagement of trans people from specific ethnic backgrounds [24].

An important factor contributing to delays in the development and responsible use of AI for trans health care is the lower continuation rate in STEM (Science, Technology, Engineering, and Mathematics) majors of trans individuals compared to their cisgender peers, despite high levels of academic ability and self-confidence [25] and the growing attempts to enhance visibility in the field [26,27]. Moreover, a lack of diverse environments and understanding of trans realities also affects medical education, where morphological diversity and realism in health care simulations are often neglected [28]. This unpreparedness can lead to challenges even in standard medical procedures such as tracheal intubation [29]. Infrastructural actions to improve trans health care globally, and specifically the possible adoption of AI solutions, should account for local realities at the national level. For instance, in Spain, where this work has been developed, significant discrepancies have been reported in the general management of trans health care [30], impacting the quality and value of the service as well as their satisfactory accessibility by the community.

In this work, we investigate the perception and use of AI in trans health care by engaging volunteer trans people in Spain through a series of focus groups (FGs).

## Methods

### Communicative Methodology and Setting Up of the FGs

In this study, we conducted FGs applying the communicative methodology [31,32], which consists of the participation of the people involved throughout the research process and joint reflection through egalitarian dialogue. These 2 aspects represent the most characteristic and innovative elements of this methodology, allowing for bridging the methodological gap and acknowledging cultural intelligence. The European Union has highlighted the communicative methodology for its excellent results in work with vulnerable populations [33].

Following this methodological approach, researchers of the Barcelona Supercomputing Center and Universitat Pompeu Fabra held a preliminary meeting with the Spanish “PRISMA” (Association for the Affective, Sexual and Gender Diversity in Science, Technology, and Innovation) association, formed by LGBTIQ+ people working in STEM, in July 2020, to define the objectives of the research and the expected results, as well as the appropriate procedures to carry out the research. The importance of including representatives of the trans community at all times of the study, including its preparation, was agreed. Accordingly, a trans woman and a trans man collaborated with the research team reviewing the different steps of the process, including the FGs schedule and the necessary materials.

### Preparation of the FG Material

Two types of materials were prepared for the FGs: a presession form and a session question guide. The presession form collected participants’ profiles, including gender assigned at birth and gender identity, allowing for a diverse representation of identities and discourses. Language, content, and communication channels were agreed by the research team and the 2 representatives of the trans community to ensure appropriate terminology and concepts. The chosen communication channels were social networks (LinkedIn, Twitter, and Instagram), informing about the study, and emails seeking maximum dissemination and participation. An example of communication is provided at the PRISMA web page [34]. In total, 16 people participated, distributed in 3 FGs.

### Step-by-Step Recruitment Process

To ensure confidentiality, PRISMA managed the recruitment process and information collection as follows: (1) the study was announced on social media (ie, LinkedIn, Twitter, Instagram), PRISMA’s private contact emails, and its website; (2) interested participants completed a presession form; (3) informed consent forms were provided to all participants before the sessions; (4) FGs were held via Zoom from March to May 2021; and (5) session transcripts were shared with participants for feedback.

### FG Execution

The FGs were conducted between March and May 2021. An informed consent form was prepared and sent to all the participants. Sessions were held via Zoom, and participants were informed that they could choose whether or not to turn on their cameras, give their real names, or specify how they wanted to be addressed, following our commitment to privacy.

In each FG, the research team asked questions prepared in advance, focusing on the impact of AI on personalized medicine and the health care of trans people. Audio was recorded following anonymity protocols and transcribed by the research team.

To ensure that participants’ voices and needs were accurately represented, the analysis and conclusions were shared with them for feedback. Participants were invited to comment, suggest editions, or add any further details, a key step to ensure that information was correctly interpreted by the research team.

### Sample

As mentioned above, 3 FGs were conducted with the participation of 16 people. Table 1 presents key variables of the sample: sex assigned at birth, current gender identity, age, province of residence, educational level attained, professional area, and the type of employment.

**Table 1.** Profiles of participants involved in the focus groups (FGs; 14 participants and 2 assisted in organizing the FGs).

	Binary sex assigned at birth	Current gender identity	Age range (years)	Place of residence	Educational level	Employment area
First FG (March 4, 2021)						
	Female	Nonbinary	20‐29	Madrid	Master	Education-training
	Female	Man	30‐39	Valencia	Master	Health and social services
	Female	Nonbinary	20‐29	Barcelona	Bachelor	Professional scientific and technical activities
	Female	Man	≤19	Toledo	Secondary	Professional scientific and technical activities
	Male	Woman	20‐29	Madrid	PhD Student	Education
	Male	Nonbinary	50‐59	Madrid	Secondary	Professional scientific and technical activities
	Male	Woman	20‐29	Barcelona	Bachelor	Unemployed
Second FG (April 8, 2021)						
	Male	Woman	20‐29	Barcelona	Bachelor	Professional scientific and technical activities
	Female	Man	30‐39	Barcelona	Vocational Training	Health and social services
	Male	Woman	50‐59	Barcelona	Postgraduate	Holistic therapies
Third FG (May 14, 2021)						
	Male	Nonbinary	50‐59	Madrid	Secondary	Professional scientific and technical activities
	Female	Man	20‐29	Castellón	Bachelor	Health and social services
	Female	Nonbinary	20‐29	Barcelona	Secondary	Professional scientific and technical activities
	Female	Nonbinary	20‐29	Valencia	Bachelor	Health and social services

Participants were selected based on convenience sampling combined with a snowball strategy. A person hired by PRISMA disseminated the research through social media to reach potential participants, prioritizing diversity in age and educational attainment. However, given the challenges posed by the postpandemic circumstances, as well as the complexity of contacting trans people due to their historical situation of social exclusion and mistrust of the scientific community, priority was given to recruit people currently undergoing a medical transition process. The FGs were conducted in Spanish and Catalan, and then they were transcribed verbatim. After that, the research team translated the selected quotes into English.

### Data Analysis

This coding was guided by the objectives of our research and also the scientific literature, introduced in the previous section, focusing on two central aspects: (1) personalized medicine and health and (2) AI and new technologies. In the first case, special attention was paid to aspects related to the training of medical professionals, medical care, and technologies associated with treatments and health care. In the second aspect, we focused on the application of AI to health care in the transition processes of trans people. This information was entered into the Atlas.ti program, which allowed us to define the codes as set out in Table 2.

**Table 2.** ATLAS.ti coding categories and definitions.

Codes	Definition
Medical care	Data and information concerning medical health care received, procedures, staff training, and treatment experiences.
Medication and medical devices	Experience regarding assigned medication, products on the market that are used for health treatment. Personal perspective on suitability for the trans population.
Education and training	Knowledge accumulated thanks to training or educational courses connected to trans health.
Data and AI^a^	AI and new technologies for trans people. Review of data, biases, and proposed improvements for the development of general AI.
AI applications	Experiences, problems, and advantages trans people experience with AI applications. Definition of improvements or tools needed for their beneficial use.

a AI: artificial intelligence.

After this coding process with Atlas.ti, we continued with the categorization process for the analysis of the fieldwork quotes extracted from the FGs. We inductively defined the categories of analysis, considering (1) the communicative orientation of the methodology, (2) findings from previous research, and (3) the participant reflections in the FGs. Following the communicative methodology, the study distinguished between barriers and solutions. Barriers are practices, dynamics, or statements that hinder social change. Solutions, on the other hand, are based on the configuration of transformative actions that enable these barriers to be overcome. Therefore, we have included within the category of barriers those attitudes, policies, and situations that hinder the medical treatment of trans people, as well as the inclusion of AI. Within solutions, we have included these practices, attitudes, or policies that are improving the medical treatment of trans people, as well as their relationship with AI. With regard to scientific literature, we took into account previous findings that determined the impact of including AI in the approach to personalized medicine, especially for trans people, summarized in the first section of this article. Finally, with regard to the review of the participant reflection from the FGs, we identified the elements (3 barriers and their respective solutions) that were repeated in the 3 FGs, elements described in Textbox 1.

Textbox 1. **Categories emerging from participant reflections: barriers identified across the 3 focus groups (FGs) and proposed solutions developed through initial data exploration.**

Barriers:

- Lack of research on the trans population. Perpetuation of biases (medication, applications, and specialized systems).
- Lack of training of health professionals and in the university system.
- Artificial intelligence with binary systems that do not consider the reality of trans people.

Solutions:

- Inclusion in medical procedures, health system for the whole population.
- Training for professionals, guided by examples that listen to the voices of and include the trans community.
- Applications with an inclusive design.

### Ethical Considerations

#### Human Participant Ethics Review

The study was performed in accordance with the ethical standards as laid down in the 1964 Declaration of Helsinki and its later amendments or comparable ethical standards. Freely given, informed consent to participate in the study was obtained from all participants. Participants responded affirmatively to providing consent in order to be engaged in the focus groups. The research has been continuously evaluated ethically by members of the trans community, PRISMA, and members of the Ethics Committee for Research into People, Society and the Environment at the Universitat Rovira i Virgili. In the first case, it is worth mentioning that 2 trans persons validated and actively participated in the process of developing the FG question guidelines. Similarly, PRISMA researchers who participated in the design and implementation of the fieldwork sought to ensure ethical criteria were met throughout the whole process. Finally, members of the Ethics Committee for Research into People, Society and the Environment at the Universitat Rovira i Virgili also reviewed the methodological approach of the study.

#### Informed Consent

Each person who participated in the FGs was sent an informed consent form via email in a Google form requesting authorization to use the information that arose from the discussion groups. This consent form specified that the criteria of the “Spanish Organic Law 1/1982 of 5 May on civil protection of the right to honor, personal and family privacy, and personal image,” and the “Spanish Organic Law 3/2018, of December 5, on personal data protection and guarantee of digital rights” would be followed. At the same time, it was also stated that participants could exercise their rights of access, rectification, limitation of processing, erasure, portability, cancellation, and revocation of the consent given at any time by writing to PRISMA with a copy of their ID card by post or by email to PRISMA’s email address for these matters: protecciondatos@prismaciencia.org.

In this consent form, the personal information requested from participants included name and surname, ID card or foreign resident ID number, date, authorization for data processing, and attendance record.

#### Privacy, Confidentiality, and Compensation

To ensure confidentiality, names have been removed, and the Results section refers only to participants and individuals. In addition, an age range has been used to avoid specifying these personal data. All personal information obtained by the research team has been stored confidentially in an electronic space that can only be accessed by members of the research team and the person hired by PRISMA who collaborated in the participant selection process.

Finally, there was no compensation of any kind to participants.

## Results

### Barriers

#### Gaps in Research and Training on Medical Treatment for Trans People

As it has been previously mentioned, there is an important gap in research on trans people’s health, which directly influences their medical treatment. This situation can drive doctors to make misdiagnoses that negatively impact patients. In the following sentence, an example of this kind of mistake is observed. One of the interviewed trans women assured that a doctor wrote in their medical record that she was a man. The justification was that it was necessary to prescribe a specific medication linked to transition hormones:

I think it was ANDROCUR...they had to tell me I had prostate cancer before they could prescribe it for me. In fact, in my medical records, it appears that I am a man and when I went to complain...they told me, “If you don’t prescribe it, and you have to go to the urologist, you’re listed as a girl. You’re not going to be treated.” In fact, I know trans guys who have problems going to the gynaecologist.

[FG2]

Similarly, participants in the FG pointed out the lack of scientific evidence and research projects that have deeply analyzed the effects of certain medicines in trans or cis people. Therefore, they confirmed that this distinction would be very useful to improve medical treatments.

I was going to add that, in many things, it is not researched whether the symptoms are different in cis women, and in the cases where it has been investigated differently between cis men and women, and it has been found. But it is not known about trans people, where we fall, whether on one side, on the other, in the middle, which could be something completely different.

[FG3]

As participants also stated, the lack of research can be connected with the training of doctors who supervise trans people’s health. Some participants affirmed they feel like a guinea pig. In this regard, they perceive that doctors most of the time are testing some medicines without knowing what their effects will be. They said that this practice creates insecurity and fear about the treatments.

We are not like guinea pigs, to see how something feels. And the first thing they prescribe, as in my case, is “Testex-100 and we try to see what happens until we reach your levels” ...When I had been taking it for 5 or 6 months, I started with the 250 and my testosterone levels were very low and they raised them every 15 days. ...then “I was going through the roof” and of course you find yourself at a moment when you say: “Is this going to be good or bad for me? How can it affect me?,” because they only tell you the changes you are going to have but not how it can affect you if you go above or below it [the desired hormonal levels].

[FG3]

According to participants, there is inequality regarding the information available about treatments. For instance, one trans woman assured that she has more knowledge of their health status than some doctors. She explained a situation where she wanted to donate blood, and the doctors refused because they said she was anemic. Based on her experience, she said they were making a mistake because, as a trans person who was undergoing hormone treatment, they could not take the same parameters as before her transition.

Sometimes we know more than the doctors who are treating us about more things. I was trying to donate blood once, and the doctor insisted that I was anaemic because they insisted on comparing my values with the men’s table. And I’m on hormone treatment, so there’s no way I was going to get it right on the men’s chart. And it’s one of the first things that they change, it’s one of the first things that they explain perfectly well, it’s one of the first things that they comment in TRANSIT [TRANSIT is a public health service of the Government of Catalonia aimed at the medical treatment of trans people from an inclusive perspective] about important medical effects.

[FG3]

#### Biases in AI

One criticism made by the trans people who participated in the research was about technological tools. Some of the participants said that these tools, based on AI, do not consider their reality and make them invisible. They also argued that this is very frequent in applications to feminize or masculinize the voice. The following quote is a graphic example of this process:

Technologies ignore us, new technologies ignore us. Not only because we are a trans population, [also for] those of us who are trans women, the double discrimination, so they ignore us. I have done voice feminisation exercises, from a masculine voice to an androgynous voice. And I have gone through voice recognition, and they have passed me as a person, as a boy. They recognised me as a boy. I found one, which appears [to have] an intermediate range, …But come on, ...I’ve gone up an octave when I did it, and now I’ve realised that I’ve gone up an octave and three notes.

[FG2]

Another statement made by some participants referred to the fact that the above-mentioned apps reproduce sexist stereotypes with very binary images that they perceive as very disconnected from the trans reality. The following dialogue, established in one of the FGs, illustrates how noninclusive these tools can be.

A: It’s a stripe like this and it’s turning pink or blue.

N: Ahhh, pink or blue, that’s interesting.

D: Exactly, I wanted to know if it is associated with categories that reflect the binary reality... that is neither pink or blue...I mean, so do you think that this for example is a form of discrimination...that is to say... up to here pink and up to here blue? What is your experience of it, what kind of sensations have you had?

A: I hated it straight away because I don’t know, I think everyone has - I’ve met cis girls who have a super voice, how do you say it?

O: masculine

A: Yes masculine.

O: In inverted commas

A: Exactly, and the opposite, so when I downloaded it and tried it out, she said it’s nonsense, I don’t know, apart from that, that it’s pink and blue.

[FG3]

### Solutions

#### Research and Training for Health Professionals Considering Trans People

Within solutions and proposals that emerged in the FGs, the relevance of training professionals aimed at listening to trans people is particularly highlighted. Trans participants proposed that this training could be undertaken in the health and medicine areas. In this regard, they highlighted the relevance of health care services in Catalonia, like TRANSIT, which is properly trained in trans health issues. One of the participants underlines that in this service, there is a close relationship with the doctors, which maintains continuous monitoring of relevant aspects of trans health.

I go through TRANSIT. Which is in Barcelona and it is true that concerning the blood analyses, well I... I have done [them] every 4 months, and whether they tell me to have done or not, I have done, I go here and ask for them and the doctor is very nice and I have no problem with her, and every time I meet her...are every 6 months and...I think you find yourself feeling delicate... above the levels you should be or your kidneys are below what they should be and these are things that you have to keep under control.

[FG3]

Research on trans health is also an issue directly linked to the training of professionals, and this is another aspect stressed by participants, who considered that this aspect would be necessary to improve. One of the transformative elements that are positively valued by trans people is the involvement of organizations in the research, like PRISMA, because they are aware of the situation of these people and work to ensure proper treatment. In accordance with the purposes of PRISMA stated on its website, it tries to promote trans-inclusive research aimed at ensuring diversity. Thus, trans people participating in the study stated that when organizations like PRISMA promote this kind of research, they perceive a sense of responsibility and confidence.

So, you have to bear in mind that when a research centre proposes to carry out a study, many people are very wary of it because it’s like, what am I going to find? I’m going to be generalised, I’m going to be mistreated, I’m going to meet someone who has no idea and I’m going to have to explain everything, I came to this because it was organised by PRISMA, and I know PRISMA and I know I can be trusted, but if it wasn’t for [PRISMA], I wouldn’t have come here to participate.

[FG2]

Similarly, participants pointed out the relevance of transferring scientific knowledge to training programs where the real needs of trans people can be visible. In that sense, the quote below illustrates the interest of trans persons to be treated by professionals whose training is based on scientific studies.

Let them do a study, with artificial intelligence or with paper, I don’t care, let them do a clear study afterwards, and with that study and with those results, go to the training of people and say, gentlemen, we need this.

[FG1]

#### Personalized Health Care Treatments for Trans People

Personalized treatment is another of the aspects underlined by participants, which means that for them precision medicine is a priority for considering trans people’s specificities in health care. It means a strong democratization of medicine, one of the issues stressed by participants. One of their recommendations was the design of an easy-reading interface where people can continuously check their health status, and, if any strange situation is identified, the person could rapidly ask for an appointment with the doctor.

I think that a method is being developed to easily perform analyses. Most medical analyses are now quite self-interpretative, maybe making a simpler interface to let you know if you have something out of level, really go to the doctor and they will automatically give you an appointment, I saw that as a great advance and not having to be dependent, in my case I am dependent on the endocrinologist to ask for the analyses.

[FG2]

These personalized treatments are directly linked to the establishment of collaborative networks among trans people themselves. For instance, a trans woman participant affirmed that a WhatsApp group was created to exchange information about health issues related to the transition process. According to her perception, this kind of interaction helps to combat several delays in the transition process caused by disinformation.

I’m in a WhatsApp group of trans people. ...You go into TRANSIT, for example, and after a month, they are already calling you, and you are already seeing that you already have hormones, you already have everything. ...people who are from other cities tell you: “first I have to go to a psychologist, then maybe I have to wait 6 months, after the psychologist I have to stay with the endocrinologist until they call me, and I think that’s like 2 years of your life lost, and I think that when we make the decision it’s because we have it very clear.”

[FG3]

#### Inclusion of the Trans Perspective in AI

Another of the concerns collected in the FGs is the inclusion of a trans perspective in the design of health-related technologies. In this regard, a participant suggested that AI should be fed with the advice of trans people. She thinks the information provided would be closer to that of a professional with no knowledge of the subject.

All the advances and everything that is carried out that concerns us; we should be there, I offer myself, but we have to be there for what concerns us. We have to come out of our vision, from our experience, everything that concerns us. Well, we have our own experience, and we don’t do things according to what other people believe about us.

[FG1]

In accordance with the importance of medicines for the trans people participating in the study, particularly if they have decided to start a biomedical transition, they opined that AI would be useful to have more accurate information about these medicines, having a better impact in their transition process.

Our imminent struggle is to have our own medicines. Because that is what A said. It is what D. says, with the side effects, since July until now I have taken 4 different brands of oestrogen...We are using medicines that are not for us, I think that artificial intelligence will be useful for that. It would be useful to help us and to have specific medication for us.

[FG1]

Another element that emerged in the FGs concerns technology with an inclusive perspective, in other words, technology that is connected to trans people’s realities. Some participants affirm that technology can advance to a nonbinary approach that contemplates gender diversity. For instance, the following quote shows how a participant explains how she can use 2 apps for training the feminization of the voice and the menstrual cycle.

I tried it, to see where my voice fell, because they always mistook me for my mother on the phone, so I’m going to try it...and that’s it. But now that you mentioned the menstrual cycle apps, there was a time when I was using a menstrual cycle app, because I noticed more or less that I had emotional cycles that more or less matched my 28-day cycles, I was using that for a while with that kind of app. It was good…it was like, hey, today I feel like crying...it gives things a bit of a new meaning because it wasn’t at all customisable, but it did help me a bit to really get to know these emotional cycles, that you had the feeling that they were really happening. And that happened until recently when my hormones became unbalanced.

[FG3]

In fact, the app that is used for training the voice is one of the technologies best evaluated by other participants in the FGs. She concluded that this app is useful for checking whether the voice suits feminization or masculinization, according to the user’s wishes.

I have an application installed on my phone which is a voice analyser. I have it because I have done exercises. It is based on exercises and when you use it you see how you are progressing and it is…it is one of the best.

[FG1]

## Discussion

In this study, we explored perceptions and uses of AI in trans health care by engaging volunteer trans participants in Spain through a series of FGs. The FGs revealed significant barriers in the use of AI for personalized medicine among trans people, including gaps in medical research and training, leading to frequent misdiagnoses and insecurity in treatment, as well as the presence of biases in health-related technologies that often fail to represent trans realities. Participants highlighted the lack of scientific studies specifically addressing trans health needs and emphasized the need for better education and awareness among health care professionals. They also pointed out that AI technologies frequently reinforce binary gender stereotypes and marginalize trans identities. In response, participants proposed solutions such as fostering research and training programs that center trans experiences, creating personalized health care tools, building support networks among trans individuals, and ensuring the inclusion of trans perspectives in the design of AI systems. They also stressed the importance of developing more tailored medicines and nonbinary-inclusive technologies to better reflect and support their diverse experiences.

Research on gender issues has documented significant social changes related to gender identity, challenging traditional gender binarism [35-37]. However, as this study highlights, it is important that technology and AI adapt to support gender diversity. An example of these changes is the depathologization of treatments addressed to trans people. In June 2019, trans and gender-diverse identities were removed from the *International Classification of Diseases, 11th Revision,* in the 72nd Assembly of the WHO [38].

Along the same lines of social change, health care has also advanced, benefiting from social advances in which technologies and AI have played an important role [9,10,39]. For example, AI-powered personalized medicine is contributing to better health monitoring [40]. The scientific literature illustrates a few examples that have been transferred to nonconforming gender individuals. One of these initiatives is the development of gender-neutral voice for digital assistants, the so-called Q app, aimed at reducing gender bias in health technologies. Similarly, in cardiology, an AI system is used to analyze variations in the electrocardiogram of individuals undergoing gender-affirming hormonal treatment [18].

Recent initiatives stress the need to operationalize AI ethics in health care through compliance mechanisms and reflective practices, with particular attention to bias, privacy, and equity. International guidelines, such as the United Nations Educational, Scientific and Cultural Organization’s “Recommendation on the Ethics of Artificial Intelligence” [41] and the OECD AI Principles [42], advocate for human rights, transparency, accountability, and inclusivity in the deployment of AI in health settings. However, research specifically examining the impact of AI on trans communities remains limited.

Existing studies highlight unique vulnerabilities in AI-mediated mental health services for trans individuals, underscoring the urgency for inclusive, rights-based reforms. For example, a 2023 UK parliamentary briefing [43] reported that AI referral chatbots increased referrals among minority groups by reducing perceived judgment, but also raised concerns about bias, privacy breaches, and harmful representations. Recently, the American College of Chest Physicians explored how AI tools like ChatGPT could serve as allies to the LGBTIQ+ community, by enhancing access to information, fostering supportive interactions, and promoting inclusivity in health care settings, emphasizing that their success depends on thoughtful design and collaboration [44]. The need for careful, inclusive AI design is further reinforced by studies on technologies such as facial recognition and automatic gender recognition that demonstrate how these systems can perpetuate and amplify societal prejudices against nonbinary and trans individuals [12]. In this context, ensuring the robust protection of trans individuals’ personal data becomes even more critical, both for current systems and as AI-driven health care technologies continue to evolve. Finally, efforts are also underway to improve electronic health records, with detailed guidance on how to better document preferred names, pronouns, gender identity, and sex assigned at birth, key changes that can significantly improve the quality of care for trans and gender-diverse patients [45]. One example is athenaOne, a cloud-based electronic health records suite that has implemented these inclusive practices [46]. Further commercial innovations are helping to enhance health care services for trans and gender-diverse communities, such as the app TranZap [47], connecting trans individuals with gender-affirming health care providers.

This study corroborates previous analyses highlighting the existing gender bias in the treatment of trans people in medicine, technology, and in the application of AI. Some of the challenges identified by the FG participants pay attention to the gender stereotyping in the applications of AI to medicine, which makes trans people invisible. Drawing on some participants in our research, this algorithmic and cognitive gender bias is also connected with the lack of training and inclusive perspective among health professionals about gender diversity.

On the other hand, findings reveal several insights that could help address some of these challenges. First, they highlight the need to incorporate the experiences of trans people, through a cocreative process [48], in the design of applications and technologies that address their health care. To this end, participants stressed the importance of training health and medical professionals from a trans-inclusive perspective. They also highlighted the importance of networking on health issues; thus, sharing information has made it easier for them to accelerate certain processes related to their transition process. Some of the participants underline the relevance of considering their own experience and knowledge in their treatments, which is often extensive due to the processes of self-education and experience of trans people. They perceived that if this information is considered, the approach to their health improves greatly.

The review of the literature and the empirical data collected allow us to propose some recommendations.

First, we advocate for digital applications designed to monitor people’s health to be developed inclusively, taking into account different target populations, and in particular trans people. Our research shows that, when effort is put into the personalization of these applications, including gender diversity, they can have a very positive impact.

Second, according to several participants, trans people’s confidence in the health system increases when actions and professionals consider their reality, that is, when their voices and their accumulated experience are listened to and not invisibilized.

Finally, the promotion of solidary networking and strategies of knowledge exchange between trans people and health professionals can make it possible to have better treatments.

These 3 principles (developing inclusive AI health applications for trans people, fostering trust through acknowledgment and cocreation, and promoting solidarity networks) are closely aligned with the goals and requirements of major governance frameworks such as the GDPR (General Data Protection Regulation) and HIPAA (Health Insurance Portability and Accountability Act). Inclusive, personalized health care design reflects GDPR’s focus on data minimization and privacy, as well as HIPAA’s emphasis on patient confidentiality. Strengthening trust through acknowledgment and cocreation aligns with GDPR’s commitment to user control and HIPAA’s support for patient empowerment. Finally, promoting solidarity networks and knowledge exchange echoes GDPR’s principles of community participation and HIPAA’s focus on collaborative care. However, it is important to note that while regulatory frameworks for AI systems, such as the EU Artificial Intelligence Act, acknowledge that AI can perpetuate and amplify biases, including in health care, they do not specifically or comprehensively address the unique forms of bias affecting trans individuals. As a result, those impacted may have to rely on national laws, which often impose a higher burden of proof and leave significant gaps in protection [49].

This study identifies several key areas for policy action. First, it is essential to mandate the inclusion of trans perspectives in the design, development, and evaluation of health care technologies, including AI-based ones. Regulatory frameworks should explicitly address algorithmic bias affecting people from diverse communities (and in particular LGBTIQ+ people), strengthening requirements for fairness, transparency, and inclusivity in a framework of equality, diversity, inclusion, and accessibility of innovations. In addition, education policies should promote mandatory training in trans health and gender diversity for medical and technological professionals. Policy makers must also support the creation of inclusive health data infrastructures that recognize gender diversity while protecting privacy. Finally, fostering community-led research initiatives can build trust and ensure that digital health innovations genuinely serve and empower trans and gender-diverse populations.

To conclude, the main limitation of our study lies in the limited generalizability of our findings, given the qualitative sample used. For future research, it would be relevant to use larger samples and quantitative techniques that would allow reaching a broader population. However, it should be noted that it is sometimes difficult to gain access to trans people and their health-related realities. For example, the lack of disaggregated data reflecting gender diversity in Spain complicates data analysis. In any case, the participation in the study of PRISMA has allowed us to access trans people with confidence in the work aimed at respecting their rights and also in the search for scientific knowledge that can improve their well-being and the development of better AI systems for health. Ultimately, this study highlights the urgent need for more inclusive research practices and data infrastructures that not only advance scientific understanding but also ensure that emerging AI technologies in health care genuinely serve and empower trans communities.

## Abbreviations

AI: artificial intelligence
*DSM*: *Diagnostic and Statistical Manual of Mental Disorders*
FG: focus group
GDPR: General Data Protection Regulation
HIPAA: Health Insurance Portability and Accountability Act
LGBTIQ+: lesbian, gay, bisexual, transgender, intersex, queer, and other identities related to affective, sexual, and gender diversity
PRISMA: Association for the Affective, Sexual and Gender Diversity in Science, Technology, and Innovation
STEM: Science, Technology, Engineering, and Mathematics
WHO: World Health Organization
